# rTgOWP1-f, a specific biomarker for *Toxoplasma gondii* oocysts

**DOI:** 10.1038/s41598-020-64590-4

**Published:** 2020-05-14

**Authors:** Susana Sousa, André Almeida, Lurdes Delgado, Antónia Conceição, Cláudia Marques, José Manuel Correia da Costa, António Castro

**Affiliations:** 1 Center for Parasite Biology and Immunology, National Institute of Health Dr Ricardo Jorge, Rua Alexandre Herculano 321, 4000-055 Porto, Portugal; 2 Center for the Study of Animal Science (CECA)/Institute for Agricultural and Agro-Alimentary Science and Technology (ICETA), University of Porto, Porto, Portugal; 3 Coimbra Polytechnic PT, Bencanta, 3045-601 Coimbra, Portugal; 4 LAQV, REQUIMTE, Department of Chemistry and Biochemistry, Faculty of Sciences, University of Porto, Porto, Portugal

**Keywords:** Biochemistry, Molecular biology

## Abstract

*Toxoplasma gondii* oocyst wall protein 1 (TgOWP1) integrates a family of seven proteins, consensually assumed as specific antigens of *Toxoplasma gondii* oocyst stage, located in the outer layer of the oocyst wall. Herein, we notice the expression of a recombinant antigen, rTgOWP1-f, derived from a fragment selected on basis of its structural homology with *Plasmodium* MSP1–19. Rabbit polyclonal antibodies anti-rTgOWP1-f evidence ability for specific identification of environmental *T. gondii* oocysts. We assume, rTgOWP1-f, as a possible biomarker of oocysts. In addition, we present findings supporting this vision, including the development of an immunodetection method for *T. gondii* oocysts identification.

## Introduction

*Toxoplasma gondii* infections are widely prevalent in humans and animals worldwide^1^. Humans become infected postnatally by ingesting tissue cysts from undercooked meat and consuming food or drink contaminated with oocysts^2–5^. The relative importance of transmission via tissue cysts versus oocysts is unknown^4^. Consensually, literature admits that oocysts can remain viable for long periods in environment and, in addition, they are resistant to chemical and physical treatment currently applied in water plants, including chlorination and ozone treatment^6,7^. However, the detection of *Toxoplasma* oocysts in water is complex, and no standardized methods are available. Recently, Heather Fritz and Patricia Conrad, 2018^8^, proposed a strategy for oocyst identification based on antibodies against a selected group of TyRP’s and TgOWP2 proteins (US 2018/0017557A1). Herein, we propose a different approach: an immunofluorescence assay based on rabbit polyclonal antibodies against a selected sequence, rTgOWP1-f. The choice of TgOWP1 as a possible biomarker for environmental oocysts was based on its location in the outer layer wall of both sporulated and unsporulated oocysts^9,10^. However, a technical constraint was clear; TgOWP1 sequence and primary structure is complex, and presents additional difficulties in the expression of a homologue recombinant antigen in *E. coli*, because its low solubility and low expression^9^. To overcome the situation, we have submitted TgOWP1 sequence at structural searches by ExPASy workstation in order to identify fragments that may constitute targets to host immunological response. Curiously, we have remarked that several sequences within TgOWP1 gene present structural homology with *Plasmodium* merozoite surface protein 1 C-terminal 19-kDa fragment (MSP1–19). This peptide is involved in the interaction of *Plasmodium* merozoites with red cells membranes, and it is highly immunogenic in malarial infections^11,12^. This finding was critical for our choice. We designed specific primers to amplify the portion of the gene coding the sequence referred as TgOWP1-f and presenting structural homology with MSP1–19. A recombinant homolog sequence was expressed in an *E. coli* vector and purified. Polyclonal antibodies against the recombinant protein, rTgOWP1-f, obtained after rabbit and mice immunization, evidence a clear-cut ability to identify *T. gondii* oocysts.

## Results

### Structural analysis of TgOWP1

**Figure 1:**
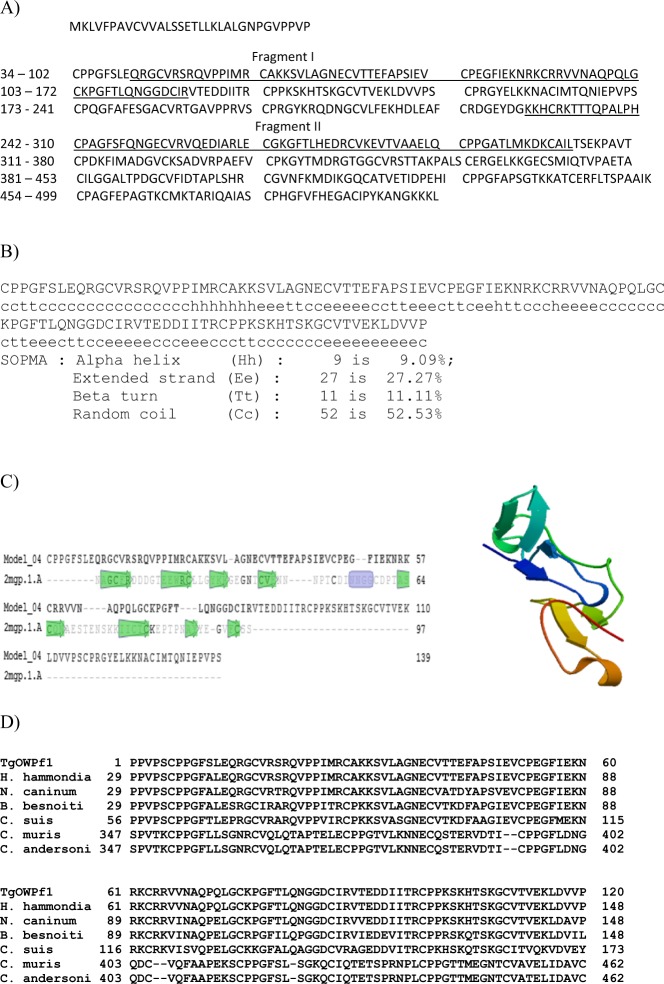
Analysis and description of rTgOWP1 fragment. **A**) Partial sequence of protein TgOWP1 and identification of the fragment TgOWP1-f; The amino-acid sequence corresponding to TgOWP1-f is identified with grey background; The sequences 34–102; 103–172; 173–241; 242–310; 311–380; 381–453; 454–499 are the TgOWP1 cysteine-rich motifs. Sequences with structural homology with merozoite surface protein I are underscored and identified as Fragment I and Fragment II. **B**) Structural data TgOWP1-f; The TgOWP1-f (Fragment I) sequence was analyzed by the SOPMA secondary structure prediction method. **C**) Structural comparison between the sequences of Fragment I and sequences from *Plasmodium* Merozoite surface protein 1. Fragment I structural comparison with sample 2mgp.1.A from MSP1–19 of *P. Yollie*; 3D ribbon image of TgOWP1-f predicted structure. **D**) Sequence alignment of TgOWP1-f of *T. gondii* (EU 851867) with H. hammondia - *Hammondia hammondi* oocyst wall protein 1 (KL 544053) fragment, N. caninun - *Neospora caninum* putative oocyst wall protein (XP 003882327) fragment, B. besnoiti - *Besnoitia besnoiti* oocyst wall protein (XP 029219539) fragment, C. suis – *Cystoisospora suis* oocyst wall protein (PHJ19967) fragment, C. muris – *Cryptosporidium muris* oocyst wall protein (XP 002140636) fragment, and C. andersoni - *Cryptosporidium andersoni* oocyst wall protein (OII76225) fragment.

TgOWP1 is a 499-amino acid protein, with a putative signal peptide sequence, followed by six type I (six-cysteine) domains and by a single four-cysteine type I domain at the C-terminus. Type II domains are absent in TgOWP1. The domain structure of TgOWP1 (Fig. 1a) was previously described^13^. Analysis of proteins containing sequences homologues to the TgOWPf with BLAST shows high identity values (> 90%) with proteins from *T. gondii* and with an oocyst wall protein of *Hammondia hammondi* (Fig. 1d). ExPASy workstation was utilized in the search for structural homologies, and highlighted the presence of two fragments with significant homology to the C-terminal sequence of *Plasmodium* merozoite surface protein I (MSP1–19) (Fig. 1c). The TgOWP1-f shows significant structural homology with *P. yoelii* MSP1–19 (sample 2mgp.1.A from ExPASy Structural database)^14^ with values of Global Model Quality Estimation (GMQE) of 0.24 and Qualitative Model Energy Analysis (QMEAN) of −3.53, and a sequence identity of 21.13% (Fig. 1c). Structural analysis of the fragment TgOWP1, by Swiss Prot Modelling, and secondary structure tools^15^, suggest that the fragment has several extended strands separated by random coils (Fig. 1b). Here, we describe a truncated protein of 120 amino acid (Fig. 1a in grey) corresponding to the second exon of the gene. The TgOWP1 fragment includes the first type I domain and the four-cysteine sequence of the second domain (Fig. 1a).

### Expression and purification of TgWOP1-f recombinant antigens

**Figure 2:**
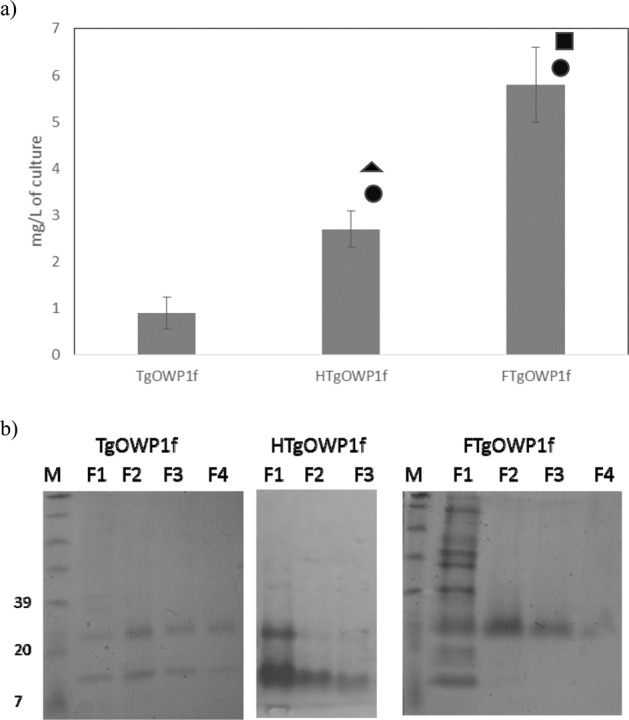
Expression of rTgOWP1-f into the vectors pQE30, pQE30H, and pQE30F. (**a**) Quantification of total protein for recombinant antigens rTgOWP1-f (0.9 mg/L), rHTgOWP1-f (2.7 mg/L), and rFTgOWP1-f (5.8 mg/L). Data represents means ± standard deviation of triplicate experiments. Circle represents statistically significant difference from TgOWP1-f; triangle represents statistically significant difference from FTgOWP1-f; square represents statistically significant difference from HTgOWP1-f. (**b**) Analysis in Tris-tricine SDS-PAGE gel of the recombinant antigens rTgOWP1-f (15 kDa), rHTgOWP1-f (16 kDa) and rFTgOWP1-f (23 kDa). Four fractions were collected during proteins elution (F1, F2, F3, and F4).

TgOWP1-f was cloned into the vectors pQE30, and pQE30H for immunological purposes^13,16^, and pQE30F^13,17^ for production purposes (Fig. 2a,b). As expected, antigens production showed a significant increase for both tags, although more important when the F-tag was used (5.8 mg / L for rFTgOWP1-f, 0.9 mg / L for rTgOWP1-f and 2.7 mg / L for rHTgOWP1-f). Purified proteins were analyzed by SDS-PAGE (Fig. 2b). Molecular weight was slightly higher than expected (15 kDa for rTgOWP1-f, 16 kDa for rHTgOWP1-f and 23 kDa for rFTgOWP1-f), probably associated to structural characteristic of TgOWP. rFTgOWP1-f antigen was used for serological assays to evaluate the presence of specific anti-TgOWP1-f antibodies.

### Evaluation of TgOWP1-f immunogenicity

**Figure 3:**
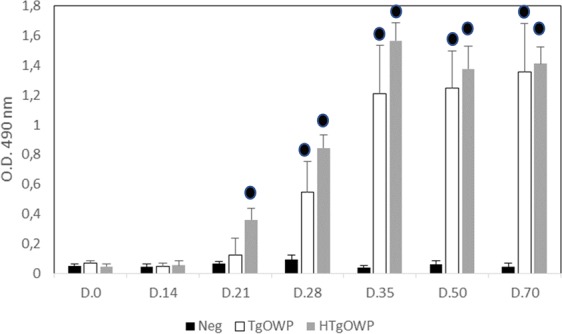
Analysis of specific anti-rTgOWP1-f IgG produced in mice upon immunization. Mice were injected IP either with 10 µg of the respective recombinant antigen (rTgOWP1-f, rHTgOWP1-f) diluted in PBS or injected with PBS (control group). Sera was collected prior to injection (D.0) and the presence of IgG against TgOWP1 was detected by ELISA using rTgOWP1-f as antigen at day 14 (D.14), 21 (D.21), 28 (D.28), 35 (D.35), 50 (D.50), and 70 (D.70) after immunization. The optical density (OD) values were set on the mean absorbance reading for 3 mice ± standard deviations. Circle represents statistically significant differences to controls.

To confirm the development of antibodies against rTgOWP1-f, mice were immunized with soluble recombinant antigen without the use of adjuvant and ELISA evaluated their immunogenicity. The presence of specific antibodies against rTgOWP1-f was detected from the 3^rd^ week onwards (Fig. 3). A plateau was reached at day 35 after injection. Both antigens are immunogenic. However, the presence of the H-tag seems to increase the consistency of the level of antibodies against rTgOWP1-f. For production of specific antibodies against TgOWP1-f in rabbits, rHTgOWP1-f was used due to the increased levels of production.

### Production of specific antibodies

**Figure 4:**
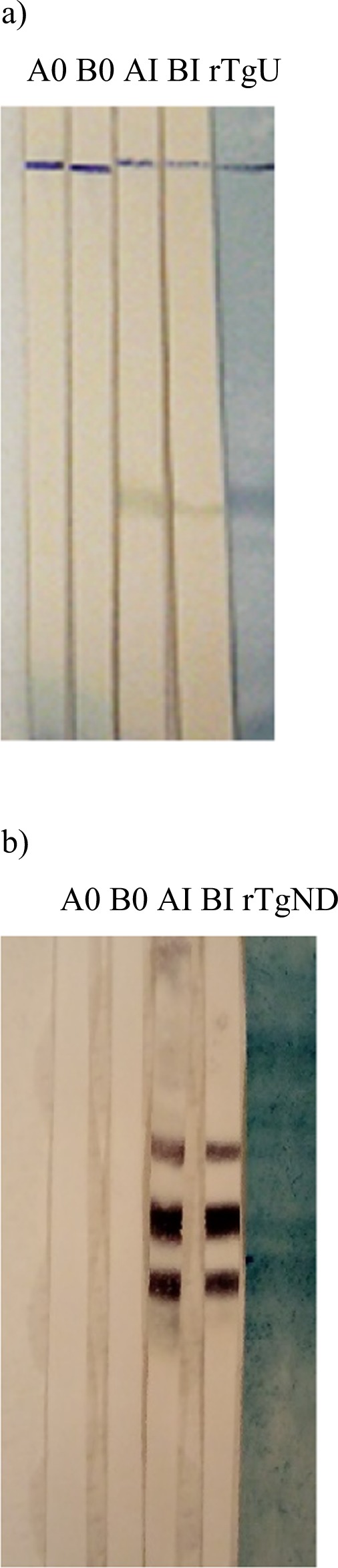
Immunoblotting with rTgOWP1-f. Rabbit sera raised against rHTgOWP1-f was evaluated using rTgOWP1-f in 8 M Urea buffer (**a**) or rTgOWP1-f obtained in native conditions (**b**). A0, B0 – Sera from rabbit A or B collected before first immunization; AI, BI – Immunosera from rabbit A or B raised against rHTgOWP1-f.

**Figure 5:**
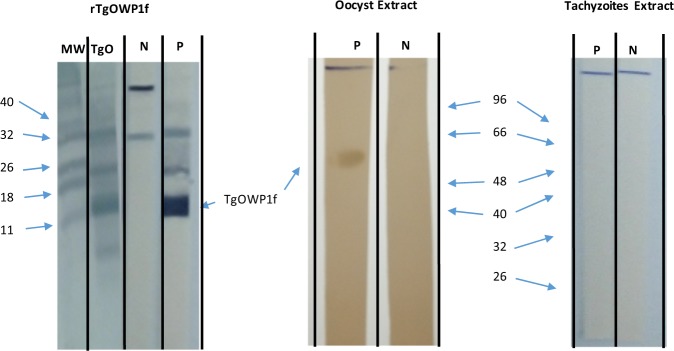
Identification of TgOWP1-f in *T. gondii* extracts. *T. gondii* oocyst and tachyzoites extract were probed with specific anti-TgOWP1-f sera (P) or pre-immune (N) sera to evaluate the presence of the antigen in the extract. MW – molecular weights; TgO – membrane with recombinant antigen rTgOWP1-f stained with Amido Schwartz; rTgOWP1-f – Immunobloting results using rTgOWP1-f antigen; Oocyst extract – Immunobloting results using oocyst extract; Tachyzoites extract – Immunobloting results using Tachyzoites extract. rTgOWP1-f, oocysts extract and tachyzoite extract were not run on the same blotting and were not probed together with the anti-TgOWP1-f sera because we believe that specific antibodies could bind mainly to the recombinant antigen, hence decreasing the signal in the oocyst extract (competition effect).

In order to evaluate the specificity of the antibodies an immunoblotting assay was done using rTgOWP1-f under native (Fig. 4b) and denaturated conditions (Fig. 4a). Under native conditions, three bands with molecular weights compatibles with a monomer, a dimer and a trimer of rTgOWP1-f were observed, suggesting that rTgOWP1-f is able to form polymeric structures. Anti-rTgOWP1-f antibody recognized the native protein in oocysts (Fig. 5). However, does not recognized the protein in *T. gondii* tachyzoites.

### Detection of *T. gondii* oocysts by immunofluorescence

Immunofluorescence assay was performed to evaluate the ability of antibodies anti-rHTgOWP1-f to detect oocysts previously treated with NaOCl, as prior described^18^. The immunofluorescence assays were done with oocysts in suspension and visualized without any fixation process. The immunoserum recognized the wall of both sporulated and unsporulated oocysts (SI – figure B and D). No significant fluorescence was observed with pre-immune and control negative serum (SI – figure E). In order to determine if the antibody is specific for oocysts, immunofluoresce assays were done with *T. gondii* tachyzoites. Antibodies anti-rHTgOWP1-f did not recognize *T. gondii* tachyzoites (SI – figure F). For detection of cross reactions, similar procedures were taken with *Cryptosporidium parvum* oocysts and *Giardia lamblia* cysts. No fluorescence was detected (SI – figure H).

## Discussion

The detection of *Toxoplasma* oocysts remains an issue for many years. Herein we describe a new recombinant TgOWP1 derived-fragment, rTgOWP1-f, as biomarker for environmental oocysts. The TgOWP1 selection was based on i) its presence in the oocyst wall protein^9^, ii) not detectable in tachyzoites^9^, iii) neither in tissue cysts^10^. Protein structure and function of TgOWPs family is not clear. Nevertheless, a potential role on the production of an extracellular matrix inducing heteropolymeric complexes stabilized by disulphide or di-tyrosine bridges has been suggested^9^. In addition, Santana *et al*., 2015 reported specific antibodies anti-TgOWP1 in naturally infected hosts^10^, and Sotiriadou and Karanis, 2008, suggested it as target gene for the detection of *T. gondii* oocysts in natural water samples^19^. The selection of this specific TgOWP1 sequence/fragment was based on its structural homology with a C-terminal sequence of *Plasmodium* merozoite surface protein I-19 (MSP1–19) and, critically, high diversity comparing with TgOWP family proteins or in similar proteins from *Cryptosporidium spp, Besnoiti spp, Hammondia hammondi* and *Neospora caninum*. Our findings indicated high immunogenicity of rTgOWP1-f and a strong ability to recognize *T. gondii* oocysts specifically. Unfortunately, *Neospora caninum* or *Hammondia hammondi* oocysts were not available to be included in the assay. The sensitivity and specificity of these antibodies is critical for the development of ImmunoMagnetic Separation/IFA assay similar to EPA Method 1623^20^. So far, three monoclonal antibodies were described: mAb 3G4^21^, mAb 4B6^22^, and MAb K8/15–15^23^. Its specificity is under scrutiny. Recently, during a survey in order to detect and quantify the presence of *T. gondii* oocysts in fresh vegetables and fruits, usually raw eaten, the antibodies against rTgOWP1-f proved to be helpfully (Marques *et al*., ongoing work). Nevertheless, its specificity is under scrutiny, and deeper investigation are warranted in order to decipher the grade of environmental *Toxoplasma* oocysts contamination.

## Methods

### Study Approval

The maintenance and care of experimental animals complied with the Portuguese and European guidelines for the human use of laboratory animals. Mice were maintained in INSA animal facilities at the Centro de Saúde Pública Doutor Gonçalves Ferreira (CGF). The ethics committee for animal experimentation (ORBEA) from CGF approved all procedures. Mice were maintained in individual cages, and they received food and water *ad libitum*. Rabbits were maintained at Escola Superior Agrária de Coimbra (ESAC) animal facilities and the ethics committee (ORBEA) from ESAC approved all procedures. Rabbits were randomly allocated in separate stables.

### Parasites

Oocysts of *T. gondii* ME49 strain (kindly provided by JP Dubey). Tachyzoites of *T. gondii* RH strain were obtained from intraperitoneal lavage from mice with 48 h of infection. Oocysts of *Cryptosporidium parvum* and cysts of *Giardia lamblia* from “Aqua-Glo G/C Direct Comprehensive Kit, Waterborne, Inc., New Orleans” were used as negative controls for immunofluorescence assays.

### *T. gondii* oocysts lysate antigen

Approximatly 10^7^ oocysts were incubated overnight at room temperature with 1% Tween 80. Oocysts were washed by centrifugation to remove Tween 80, ressuspended in 200 µl of SDS-PAGE sample buffer, boiled for 15 minutes and stored at −20 °C until use.

### Preparation of *T. gondii* genomic DNA

Tachyzoites from the *Toxoplasma gondii* RH strain were washed three times in sterile saline solution and recovered by centrifugation at 1062 g for 10 minutes. Parasite suspension was adjusted to 1 × 10^8^ tachyzoites/ml in sterile saline solution. The *T. gondii* genomic DNA was extracted with QIAamp DNA Mini Kit (Qiagen), according to the manufacturer’s instructions.

### Preparation of TgOWP1-f DNA

The amplification of a portion of *TgOWP1* gene and the expression of a truncated protein was the strategy used to overcome the presence of several introns. The *TgOWP1* gene possesses three introns of 206, 85 and 393 nucleotides length, and includes an amino-terminal intron that delineates a putative signal peptide sequence present on a short exon. Primers were designed in order to amplify the entirely second exon and introducing restriction enzyme recognition sites. Primers sequence were: TgOWP_SacI 5′-TGTGCCTGTGTGAGCTCCCTCCTGTG-3′ and TgOWP_KpnI: 5′-TGATGCGCGGTACCCTAGGGAACGAC-3′. TgOWP fragment was amplified by PCR using 10 µl of *T. gondii* DNA sample, 2 µl of 25 mM MgCl_2_, 1 µl of dNTPs mix (1 mM), 10 pmol of each primer, 5 µl of Taq buffer (Thermo Scientific), and ultra-pure water to complete a 50 µl volume. PCR amplification was performed with an initial denaturation step (4 min at 95 °C), followed by 30 cycles of denaturation (30 s at 95 °C), annealing (30 s at 50 °C), and extension (1 min at 72 °C). The program included a final extension step of 7 min at 72 °C, and it was performed in a C1000 Touch Thermal Cycler (Bio-Rad). PCR fragment was separated in LMAg and isolated with illustra GFX PCR DNA & Gel Band Purification Kit (GE Healthcare) according to the manufacturer’s instructions.

### Subcloning of TgOWP1-f

The TgOWP1 fragment was subcloned in pGEMT easy vector (Promega) and transformed into *E. coli* XL1 Blue cells. The pGEMT – TgOWP plasmid was isolated with Wizard Plus SV Miniprep DNA Purification System Kit (Promega) and sequenced at Eurofins (Germany) sequencing services. The fragment was isolated from pGEM-TgOWP plasmid upon digestion with *SacI* (Promega) and *KpnI* (Promega) restriction enzymes and subcloned in the vector pQE30 (Qiagen), the vector pQE30 with the H-tag, and the vector pQE30 with the F-tag^13,16,17^.

### Expression and purification of TgOWP1 proteins in *E. coli*

*E. coli* were cultivated in LB medium supplemented with ampicillin (100 µg/ml) and kanamycin (50 µg/ml) at 37 °C. The culture was induced with 1 mM of isopropyl-β-D-1-thiogalactopyranoside (IPTG) for 5 h at 37 °C^17^. Cells were harvested by centrifugation at 4000 g during 15 minutes at 4 °C, and the pellet was lysed with 8 M urea, pH 8.0, overnight at room temperature and constant stirring of 150 rpm. Cell extracts were centrifuged at 10.000 g for 20 minutes and resulting supernatant and pellet were collected separately for further analyses. The supernatant was applied into a Ni-NTA column (Qiagen) pre-equilibrated with 8 M urea, pH 8.0. Ni-NTA purification was conducted according to the manufacturer’s instructions^24^, and the protein elution performed by a pH decrease from 8.0 to 4.5. SDS-PAGE gels stained with Coomassie-blue dye was used to analyze the purity of collected fractions from Ni-NTA chromatography. Protein concentration of each collected fraction was determined by Bradford assay.

### Immunization of mice

In order to remove endotoxins, rTgOWP1-f and rHTgOWP1-f were treated with Polymixin B beads (Pierce) according to manufacturer’s protocol. Finally, filtered through a 0.22 µm filter (Millipore) and stored at −20 °C until use. Quantification of protein was performed according to the Bradford Method (Bio-Rad). Immunization of BALB/c mice (10 to 12 weeks old) after inoculation with 10 µg of recombinant antigen in a 200 µl final volume (in apirogenic PBS), via intraperitoneal, during 35 days (five boosts at days 0, 14, 21, 28, and 35). A group including three mice was performed for each of both recombinant antigens. The experiment includes a control group inoculated with PBS. Blood samples collected in tail vein were taken prior to immunization and at days 14, 21, 28, 35, 50 and 70, and preserved at −20 °C.

### White rabbit immunization

The recombinant antigen rTgOWP-f was dialyzed against phosphate buffer solution and concentrated using Centricon 3 (Millipore) to a final concentration of 1 mg/ml, and sterilized using a 0.22 µm filter (Millipore). Aliquots were stored at −20 °C. A New Zealand white rabbit was immunized, subcutaneously, once a day, during two weeks, with 100 µg of rTgOWP1-f in 500 µl of *inoculum*, using Alum as adjuvant. A blood sample was collected from the ear, once a month, and preserved at −20 °C.

### Immunofluorescence

*T. gondii* ME49 oocysts concentrated at 1×10^4^/ml were treated with bleach (NaOCl) diluted in water to obtain final concentration ranging at 0.5, 50 and 500 mg of NaOCl per liter during 20 minutes at room temperature^18^. A solution of 10% sodium thiosulfate was added to neutralize any residual NaOCl. Oocysts were recovered by centrifugation for 10 minutes at 1000 g, ressuspended in 1 ml of 1% Tween 80 and washed three times in PBS - Tween 20 0,3% (PBST) for 10 minutes at 1000 g. Oocysts were ressuspended in diluted (1:40) rTgOWP1-f-immunized sera or negative serum and incubated at 37 °C for 1 h 30 m. Samples were washed twice in PBS, ressuspended in PBST containing anti-rabbit Ig-FITC diluted at 1:100, and incubated at 37 °C for 1 h. Oocysts were visualized in a Nikon fluorescence microscope (Nikon EFD-3). Images with 400-x magnification were done using a PowerShot A630 digital camera (Canon).

### Enzyme-linked immunosorbent assay

The ELISA tests were performed using 96-well Nunc (Roskilde, Denmark) plates coated with 10 µg/ml rTgOWP1-f in 0.1 M carbonate/bicarbonate buffer, pH 9.6, by incubation overnight at 4 °C in a moist atmosphere. The plates were washed twice in PBS-T. The remaining binding sites were blocked with 0.3% (w/v) gelatin in PBS-T for 1 hour at 37 °C. The sera of BALB/c mice were diluted to 1:500 in PBS-T and incubated for 2 hours at 37 °C. The wells were washed three times with PBS-T. Protein A horseradish peroxidase conjugate (Bio-Rad) was diluted at 1: 2000 in PBS-T and incubated for 2 h at 37 °C. The wells were washed three times with PBS-T and developed with o-phenylenediamine (OPD) for 15 minutes at 37 °C. Reaction was stopped with HCl 3 M and absorbance was measured at 490 nm.

### Western blot

SDS-PAGE was carried out using SE 250 Mini-Vertical unit (GE Biosciences). Tris-tricine gel^25^ was loaded with 100 µg of rTgOWP1-f or 1 mg of *T. gondii* tachyzoites extract (single well comb). Oocysts lysate antigen (~2 ×10^6^ oocysts) was loaded in a single well (10-well). After electrophoresis in 15% SDS-PAGE Tris-tricine gel and transferred to nitrocellulose membranes using a sandwich system using a T22 mini tank transfer unit (GE Biosciences). For rTgOWP1-f and *T. gondii* tachyzoites antigens a strip containing the protein marker and part of the antigen was cut from both nitrocellulose membranes and stained with Amido Schwartz reagent. The rest of the membranes were saturated with 5% PBS-milk solution for 1 h at room temperature. In the case of *T. gondii* oocysts antigens each single lane was cut and saturated (lane containing molecular weights was attained with Amido Schwatz). Membrane strips were incubated with rabbit anti-rHTgOWP1-f, mice anti-rTgOWP1-f, or pre-immune sera overnight at 4 °C. Protein G-peroxidase and anti-mouse IgG (H + L)-HRP (Bio-Rad) were used as conjugate for rabbit and mice sera respectively, and 4-chloro-1-naphthol in cold methanol, PBS and hydrogen peroxide for protein detection^17^.

### Data analysis

Data analysis was performed using the statistical analysis software SPSS v. 15.0 (SPSS Inc). Differences between arithmetic means were evaluated by Student´s *t*-test. Differences with a confidence interval of 95% or higher were considered statistically significant (*P* ≤ 0.05).

## Supplementary information

Supplementary Material.

## Data Availability

The datasets used and analysed in the current study are available from the corresponding author in response to reasonable requests.
