# Supplementary material for: rTgOWP1-f, a specific biomarker for Toxoplasma gondii oocysts
Source: Sci Rep. 2020 May 14;10:7947. doi: 10.1038/s41598-020-64590-4 (PMC7224171; doi:10.1038/s41598-020-64590-4)
Supplement: Supplementary file 1 — Supplementary Material. [file 41598_2020_64590_MOESM1_ESM.pdf]

rTgOWP1-f, a specific biomarker for *Toxoplasma gondii* oocysts.

Susana Sousa[a,b,d], André Almeida[b], Lurdes Delgado[a], Antónia Conceição[c], Cláudia Marques[a,b], José Manuel Correia da Costa[a,b], António Castro[a,b]

[a]Center for Parasite Biology and Immunology, National Institute of Health Dr Ricardo Jorge, Rua Alexandre Herculano 321, 4000-055 Porto, Portugal

[b]Center for the Study of Animal Science (CECA)/Institute for Agricultural and Agro-Alimentary Science and Technology (ICETA), University of Porto, Portugal

[c]Coimbra Polytechnic PT, Bencanta, 3045-601 Coimbra, Portugal

[d]LAQV, REQUIMTE, Department of Chemistry and Biochemistry, Faculty of Sciences, University of Porto, Portugal

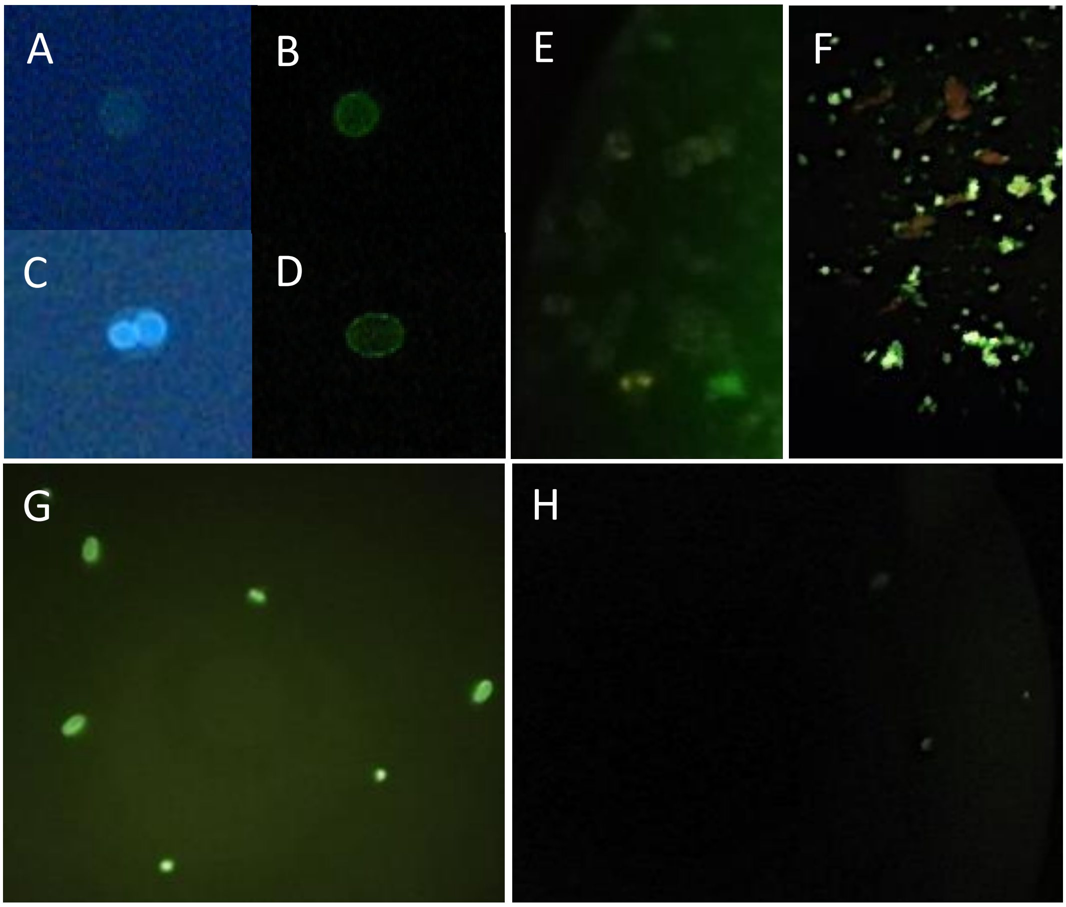

Figure SI1: Immunofluorescence assays to evaluate the specificity of the anti-rHTgOWP1-f rabbit serum against *T. gondii* unsporulated oocysts (B), *T. gondii* sporulated oocysts (D), *T. gondii* tachyzoites (F), and *Cryptosporidium parvum* oocysts and *Giardia lamblia* cysts (H). Rabbit pre-immune serum and *T. gondii* oocysts (E). *Cryptosporidium parvum* oocysts and *Giardia lamblia* cysts labelled with *Cryptosporidium* and *Giardia* specific antibody "Aqua-Glo G/C Direct Comprehensive Kit, Waterborne, Inc., New Orleans" (G). Natural auto-fluorescence *T. gondii* unsporulated oocyst (A). Natural auto-fluorescence *T. gondii* sporulated oocyst (C). Images with 400-x magnification.
